# The Little Sister

**Published:** 1857-05

**Authors:** 


### "THE LITTLE SISTER."

In reading, a few days since, the Nashville Journal, I was rather struck with the editorial devoted to the "Little Sister," down here in Atlanta; and the impression made on my mind was , that our matronly sister up there had began to experience something of the same feeling that.one of old Father Jacob's wives had for the other.

While this Sister *was* a *little Sister*, Nashville was very clever and courteous—sent down one of her Faculty to help her into life. But as she grew into womanhood and began to have admirers, "the green-eyed monster" begins to whisper *rivalry*, then comes a change in the views of the Doctors in the Capital of Tennessee.

The "little Sister" is now the mother of children, and bids fair to have a pretty numerous progeny, and to Nashville, mirabile dictu! a good many ugly marks become apparent.

The tone of the article referred to, is to my mind rather two-sided; it seems to be very friendly, yea, rather affectionate, yet it must be distinctly understood, that they regard it a terrible concern—an unprecedented innovation. We might say, in the language of Holy Writ, "Have no fellowship with the unfruitful works of darkness." If this Atlanta girl is so naughty, why this warm friendship. A short chapter on *consistency* might be read with benefit.

But let us look at these ugly marks on our little sister, that excite Nashville to such dolorous effervescing. What is the most serious defect? After all the wailing, there appears but one failing—she brings her children too soon; if she did not do that, Nashville could always hope to be up to her; but as it is, better kill her off, lest Tennessee might suffer. Good policy; keep a sharp lookout, and Nashville may discover a spot on some of the other Sisters; and if she could only keep up the slaughter, the peculiar propensity of this Sister might be fully gratified. Strange indeed the effect of lime-stone water!!

Mr. Editor, pray whisper it in the ear of Nashville, *that it is late in the day* for a question of TIME for *graduation* to be raised in *our* Medical Schools. This is a practical age, and we must look at things as *they are* and not as they might be.

How much time does Atlanta dock from the usual course? None, I answer. But Nashville says, *six months*. Wonderful!! A Doctor is made down in Atlanta *six whole* months before Nashville gets a chance at him. Well, that's very bad, certainly, for Nashville; but whether any body else is hurt by the operation, it doth not yet appear.

What is the RULE advertised by all our Colleges, as to the TIME of study? THREE YEARS. That's the law—a very good one. I wonder if Nashville has observed it. She professes to require the full time, so she publishes to the world. Now did one of her professors ever ask a student if he had rounded out the three year's study? We can refer her to some of her graduates who lacked not *six months*, but *three times* six months. Nor is Nashville singular in this; in every College that I am acquainted with, they put them through on the shortest possible notice, and with very little ceremony. A chum of mine shouldered a sheep-skin at the end of fourteen months, from the most noted college in the United States. And I am a competent witness to the fact, that he read as little as any mortal man could well manage to read in the short space allowed him. If ever he read a book clear through, his best friends were never able to tell what book it was. Nor is this an isolated case. Every physician knows that the question of *time*, with Medical Colleges in this country has ceased to be mooted, if ever it was. So let those who live in glass houses be careful how they pitch rocks about.

I am in no way connected with the Atlanta Medical College, and never expect to be, but as a medical man, I wish to see justice done her. She has fought her way through difficulties, which would have proved invincible to any but the most energetic and enterprising of the profession ; and now that these indefatigable medical soldiers have accomplished their object, and planted a successful College, which is indeed an ornament to the State and to the profession, I would see them rewarded by the approbation of their professional brethren. They richly deserve it. I know the Faculty to be composed of men of science, character and talent. They have been particular in their examinations, and the Alumni of this Institution will show that the standard of medical education has not been lowered by this School. Until this can be shown, the cry of a Summer School is not in order. It is what we need ; what the South ought to have.

If the question of *time* is raised, let the College without sin cast the first stone: if so, not many rocks will fall on Atlanta's head.

MEDICUS.

[A Medical friend has furnished us the foregoing communication, which, for sufficient reasons, at his request, is published without his name attached, and in this connection, we would commend to the Nashville School, Esop's account of the " Crab and her mother."

Said an old Crab to a young one " Why do you walk so crooked child? walk straight!" "Mother," said the young Crab, " show me the way, will you? and when I see you taking a straight course, I will try and follow."]

